# Risk Prediction of Death in Inpatient Adults With COVID-19 from Mexico

**DOI:** 10.21203/rs.3.rs-996535/v1

**Published:** 2021-11-02

**Authors:** Ruth Aralí Martínez-Vega, Wang Jing, Ana Maria Ortega-Villa, Oscar Manuel Delgado-Cuellar, Victor Alonso Hernández-Hernández, Julio Cesar Jan-Gómez, Héctor Armando Rincón-León, Paul Constantino-Santiesteban, Mariana Piedad García-Guerra, José Hiram Cetina-Díaz, José Manuel Pérez-Tirado, Omar Gómez-Cruz, Irma Yvonne Amaya-Larios, Jose Ramos-Castañeda, Sepúlveda-Delgado Jesús

**Affiliations:** Universidad de Santander; Frederick National Laboratory for Cancer Research; National Institute of Allergy and Infectious Diseases; Hospital General de Zona No.1 Nueva Frontera; Hospital General de Zona No.1 Nueva Frontera; Hospital General de Zona No.1 Nueva Frontera; Instituto Mexicano del Seguro Social; Hospital General de Zona No.1 Nueva Frontera; Hospital Regional de Alta Especialidad Ciudad Salud; Hospital Regional de Alta Especialidad Ciudad Salud; Hospital Regional de Alta Especialidad Ciudad Salud; Hospital General Tapachula; Centro Educativo de Humanidades; Instituto Nacional de Salud Pública; Hospital Regional de Alta Especialidad Ciudad Salud

**Keywords:** COVID-19, Hospitalized adult, Risk Factors, Mortality, Chronic Diseases

## Abstract

**Background:**

There is substantial variation in COVID-19 lethality across countries. In addition, in countries with populations with extreme economic inequalities, such as Mexico, there are regional and local differences in risk factors for COVID-19 death. The goal of this study was to test the hypothesis that the risk of death in Mexican COVID-19 patients was associated with the time between symptom onset and hospitalization and/or with the healthcare site. Also, death prognostic models were developed.

**Methods:**

The study included two COVID-19 inpatient cohorts, one prospective and one retrospective from Chiapas, Mexico. Demographic, clinical and laboratory variables were collected, and the diagnosis of SARS-CoV-2 infection was performed using RT-qPCR in samples collected seven days since symptom onset. The 30-day mortality, since symptom onset, was the outcome, and clinical variables at the first 48 hours of hospitalization were independent factors. Multivariate logistic regression analyses were conducted.

**Results:**

Of the 392 patients included, 233 died (59.4%). The time between symptom onset and hospitalization, the healthcare site and sex were not related to the 30-day mortality. Three death prognostic models were developed (AUC between 0.726 and 0.807). Age, LDH, AST, and lymphocyte count were included in all models, OSI-WHO Classification (Non-invasive ventilation or high-flow oxygen, and mechanical ventilation with or without organ support/ECMO) and leukocyte count in two models, and diabetes and diarrhea in one model.

**Conclusion:**

The population evaluated had underlying deteriorated health before COVID-19 compared with regional and country population. The factors that determine the COVID-19 mortality risk in a relatively healthy population are sex, age and comorbidities. However, as this study shows, when populations have underlying poor health, some of these factors lose their associations with mortality risk, and others become more important.

## Introduction

The COVID-19 pandemic has imposed a significant challenge on national health systems at all levels. Although much information has been generated on the risk factors associated with the development of severe illness and death due to COVID-19, the crucial challenge for the health systems is to avoid or decrease severe cases and mortality at the individual level.

There is substantial variation in lethality across countries. By the end of March 2021, the proportion of deaths per COVID-19 reported worldwide was close to 2.2%. Four out of the 12 countries that accounted for the most cases are located in the Americas, all with lethality near the worldwide average, USA (1.8%), Brazil (2.5%), Argentina (2.4%), and Colombia (2.6%). In contrast, during the same period, in Mexico the lethality was 9.0%, followed by Ecuador (5.2%), and Bolivia (4.5%)(1). Contrasting the number of tests per 1000 inhabitants and the COVID-19 lethality may suggest that the under-registration of total cases due to in country diagnostic test policies, may cause an over-registration of lethality since some of the countries that have the highest proportions of deaths per reported case of COVID-19 are those that apply fewer diagnostic tests per 1,000 inhabitants(2). For example, in the same time period the cumulative number of tests per 1,000 inhabitants was 1,095 in the USA, 239 in Colombia, 59 in Ecuador, and 42 in Mexico(3). Notwithstanding, in countries with populations with extreme economic inequalities, such as Mexico, differences in risk factors appear to be similar to those reported worldwide(4, 5).

There are similarities in the factors associated with severe illness and death throughout studies carried out globally(6, 7), for example age, sex(8), and comorbidities such as diabetes, high blood pressure, and obesity(9). Systemic inflammation markers (e.g. increase in IL-6 or C-reactive protein), and metabolic markers (e.g. elevated ferritin, D-dimer, Lactate dehydrogenase, and creatinine) have also been associated with severe illness and death(10–12). Although various genetic markers have been associated with COVID-19 severity(13, 14), other variables like timeliness or quality of treatment(15) could influence the infection outcome. To the best of our knowledge, the latter has not been studied in detail. We analyzed a cohort of patients with a virologically confirmed diagnosis of COVID-19 in two secondary care centers in the state of Chiapas, Mexico, between March and September 2020. Our goal was to test the hypothesis that the deaths of COVID-19 patients are associated with time between symptom onset and hospitalization and/or with the site of admission.

## Methods

### Study population and COVID-19 Tapachula study design

This was an analysis of the study, *“Clinical, genomic*, *transcriptomic, proteomic and metabolomic characterization of SARS-CoV-2 infection in Mexicans from the coastal region of the state of Chiapas” (Short name: COVID-19 Tapachula”)* performed in the state of Chiapas, México between March and December 2020. The aim of the study was to characterize the clinical, biochemical, and genetic factors related to the disease progression of COVID-19 patients in the region. The study sites were located in Tapachula, the second most populated municipality of Chiapas, located 23 km north of the border with Guatemala. These sites provided attention to COVID-19 patients in the coast region of Chiapas, through two secondary-care hospitals.

The study included two cohorts, one prospective and one retrospective. The prospective cohort was enrolled at the *“Clínica de enfermedades respiratorias COVID-19, Instituto de Seguridad Social de los Trabajadores del Estado de Chiapas (ISSTECH)”,* a secondary-care facility that provided attention to both insured and non-insured COVID-19 patients. The retrospective cohort was enrolled at the *“Hospital General de Zona No. 1 Nueva Frontera, Instituto Mexicano del Seguro Social (IMSS), Tapachula”,* that provided attention to COVID-19 patients who were employed and insured.

The prospective cohort enrolled individuals who sought care for respiratory symptoms in the previous 7 days who fulfilled the following eligibility criteria: 1) Met the operational definition of a suspected case of COVID-19 in accordance with WHO/PAHO, for the presence of sudden onset of fever and cough; or sudden onset of three or more of the following symptoms: fever, cough, weakness or fatigue, headache, myalgia, sore throat, nasal cold, dyspnea, anorexia, nausea, vomiting, diarrhea or altered mental state; 2) had an available nasopharyngeal and/or pharyngeal swab for confirmation of SARS-CoV-2 infection; and 3) agreed to participate through informed consent form. Subjects with negative RT-qPCR from pharyngeal and/or nasopharyngeal swab were excluded from the final analysis. Clinical and biochemical data were collected through case report form (CRF).

The retrospective cohort enrolled patients who sought care for respiratory symptoms in the previous 7 days, who fulfilled the following eligibility criteria: 1) Met the operational definition of a suspected case of COVID-19 in accordance with WHO/PAHO; 2) had an available molecular test for confirmation of SARS-CoV-2 infection; 3) had available laboratory tests in the first 24 hours after hospital admission; and 4) had available clinical and biochemical data of admission and hospital stay. Subjects with concomitant confirmed influenza infection, and subjects with negative SARS-CoV-2 RT-qPCR were excluded. Clinical and biochemical data was collected retrospectively.

### Confirmation of SARS-CoV-2 infection

The diagnosis of SARS-CoV-2 infection of the patients enrolled was performed with the CDC 2019-Novel Coronavirus (2019-nCoV) RT-qPCR following manufacturer indications, from pharyngeal and nasopharyngeal swabs collected seven days since symptom onset, with the modification published by Corman V.M et.al(16).

### Classification of severity of COVID-19

Disease severity (maximum score reached for each patient in the first 48 hours after admission ) was defined by a modification of the ordinal scale of clinical improvement of the World Health Organization (OSI-WHO), which classifies severity of COVID-19 in eight categories(17): 1, ambulatory without limitation of activities; 2, ambulatory with limitation of activities, home oxygen requirement, or both; 3, hospitalized, does not require supplemental oxygen and no longer requires ongoing medical attention (used if hospitalization was extended for infection control reasons); 4, hospitalized, not requiring supplemental oxygen but requiring continuous medical care (COVID-19 related or other medical conditions); 5, hospitalized, requiring any supplemental oxygen; 6, hospitalized, requiring non-invasive ventilation or the use of high flow oxygen devices; 7a, hospitalized, receiving invasive mechanical ventilation; 7b, invasive mechanical ventilation plus extracorporeal membrane oxygenation (ECMO); and 8, death. The ordinal score was calculated from CRF records (prospective cohort), or electronic records (retrospective cohort). We only considered categories 4 trough 7b because the participants were inpatients and alive at baseline.

### Clinical and Laboratory variables

Clinical and biochemical data of enrolled subjects was collected from clinical records in CRF by sub-investigators at each site. The following data were collected: date of symptoms onset, main symptoms before hospital admission, symptoms at hospital admission, comorbidities, and OSI-WHO at hospital admission. Complete blood count (CBC) and blood chemistry including glucose, urea, ureic nitrogen, creatinine, serum electrolytes, and complete liver function tests were collected within 24 hours of hospital admission. The primary outcome considered for this study was 30-day mortality since symptom onset. For participants who survived and were discharged before day 30, the outcome was obtained through phone call.

### Statistical Methods

Descriptive statistics of baseline biomarkers were calculated overall or by patient groups. To assess the difference between patient groups, Mann-Whitney, and Chi-square or Fisher’s tests were used when appropriate. No multiplicity adjustments were performed. To investigate baseline biomarkers as predictors of risk of death due to COVID-19, univariate and multivariate logistic regressions were fitted using a training set (70% of the dataset – stratified by site). Model validation was performed using a test set (30% remaining). Statistically significant features at *α* =0.05 in the univariate logistic regression analysis were included in a stepwise model selection procedure minimizing either the AIC (Akaike information criterion) or BIC (Bayesian information criterion). The study site was forced to be always included in the stepwise selection process. Selected logistic regression models with similar model assessment criteria were further evaluated on the training set with leave-one-out cross validation, and test set using ROC curves (R pROC package). Sensitivity, specificity, PPV, and NPV of the models were compared. All three models were fitted on the entire dataset to obtain final estimates of risk of death. Analyses were conducted using R version 4.0.2.

## Results

Of the 392 COVID-19 patients included during the study period, 233 died (59.4%). The majority of patients were hospitalized at the IMSS (82.4%), 67% were male, the median age was 60 years (range:18–96), the median time between symptom onset and hospital admission was 8 days (range:1–30), and the median hospitalization stay was 9 days (range:0–56), 45.2% of patients were in the fifth category of OSI-WHO (Oxygen by mask or nasal prongs), and approximately 7% of patients had mechanical ventilation or ventilation with organ support/ECMO each (Table 1).

Fever, cough and dyspnea were the most frequent symptoms, followed by nonspecific symptoms like myalgia, arthralgia, and headache. Upper respiratory symptoms including odynophagia, rhinorrhea, dysgeusia and anosmia were observed less frequently. Symptoms such as abdominal pain, hyporexia, vomit, nausea, dizziness, conjunctivitis, prechordalgia, polypnea, and cyanosis were observed in less than 10% of participants. Hypertension was the most frequent comorbidity, followed by diabetes mellitus and chronical kidney disease. Conditions such as gastrointestinal (4.3%), lung (3.6%), cardiovascular (3.6%), neurological (1.3%), and endocrine diseases (1 %), cancer (2%) and stroke (1.5%) were infrequent (Table 1). The leukocyte count geometric mean was 11.9×10^3^/cc with predominance of neutrophils, creatinine and lactate dehydrogenase (LDH) geometric means were above normal values with 1.3 mg/ml and 515 UI/L, respectively (Table 1). During hospitalization, most patients were treated with antibiotics, while steroids were only used in 20.2% of the patients (Table 2).

In the univariate analysis, a significant difference between patients who died and those who survived was observed for the following variables: site, age, OSI-WHO classification, diabetes, and symptoms such as fever, myalgia, diarrhea, malaise, and cyanosis (Table 1). In addition, the red cell distribution width, leukocyte count, neutrophil count, prothrombin time, international normalized ratio (INR), glycemia, serum urea, serum creatinine, and LDH geometric means were higher in patients who died. Conversely, the lymphocyte count and aspartate aminotransferase (AST) geometric means were lower in this group (Table 2).

**Figure 1:**
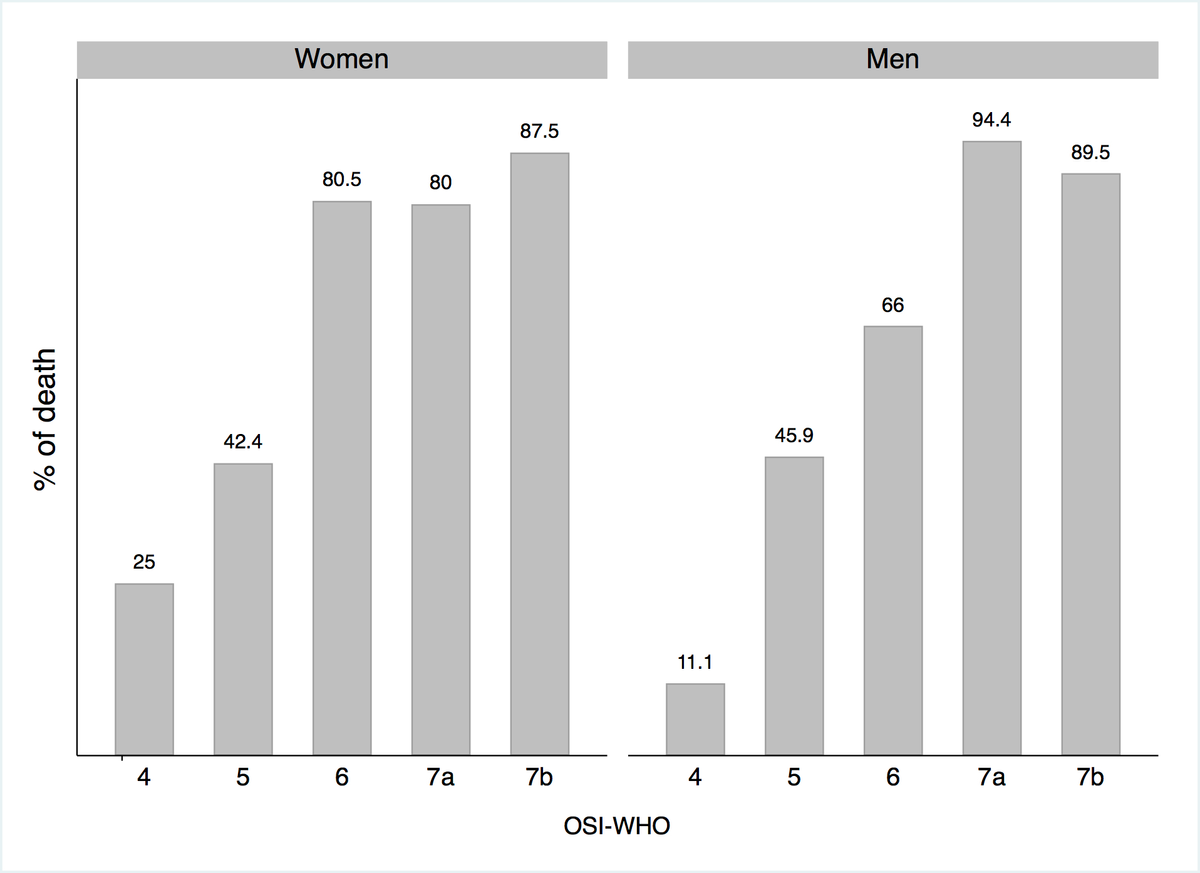
Percentage of death by OSI-WHO classification and sex (n=392).

The death frequency was similar by sex (*p*-value=0.943) (Table 1, Figure 1). Malaise, polypnea and cyanosis were more frequent in women (*p*<0.05), while neutrophil count (*p*=0.035), Neutrophil/Lymphocyte (NT/LY) ratio (*p*<0.001), urea (*p*=0.01), creatinine (*p*=0.003), total bilirubin (*p*<0.001), the aminotransferases (*p*<0.001), and the potassium (*p*=0.02) geometric means were lower in women (Supplementary Table 1). Patients of this clinical cohort had a greater diabetes prevalence than the overall Mexican and Chiapas populations, and men had a greater hypertension prevalence (Supplementary Table 2).

The numbers above the bars represent the percentage of participants who died in each OSI-WHO classification group by sex. The percentage of death increases according to OSI-WHO classification in women and men.

The time between symptom onset and admission were not related to death (*p*=0.581), with median time between symptom onset and admission of 8 days in survived patients, and 7 days in participants who died. However, the lethality was different between the sites (*p*=0.032), and there was a significant difference observed in OSI/WHO classification between sites (*p*=<0.001) (Supplementary Table 3).

Three multivariate logistic regression models were developed to predict death (training set, n=276). Age, diarrhea, LDH, AST, and the lymphocyte count were present in the three models, while OSI-WHO classification, leukocyte count and NT/LYN ratio were variables selected in two models, and diabetes, platelet count and red cell distribution only selected in one model (Table 3).

**Figure 2:**
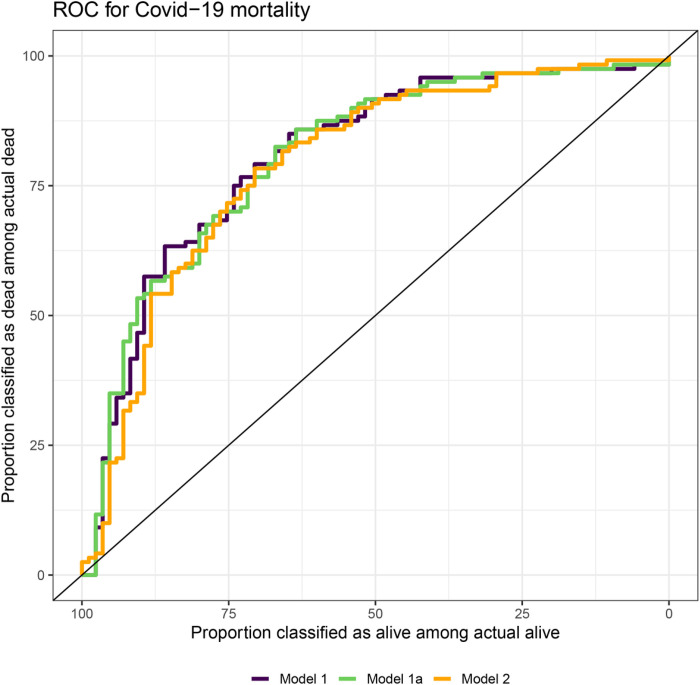
ROC for COVID-19 mortality in the Training Set

The M1a model had the highest sensitivity in the training set (n=276), however the AUCs (Figure 2) were similar among the three models (≈0.8). The M1 model had the highest sensitivity in the test set (n=116) with no difference in the AUC among the three models (Table 4). Focusing on M1, we found an association between the OSI-WHO classification and the risk of death, with higher odds of a participant dying for participants wit non-invasive ventilation or high-flow oxygen, intubation or on mechanical ventilation, and mechanical ventilation or ECMO (when compared to no oxygen therapy). We found higher odds of death for participants with diabetes mellitus, increases in Log2 LDH, and Log2 Leukocyte count, and lower odds of death with increases in in Log2 AST and Log2 Lymphocytes (Table 3).

## Discussion

COVID-19 is a disease that can have a fatal outcome(8). Lethality in hospitalized patients depends on features, including viral (infection rate, viral load, virulence), individual (genetic factors, age, sex, race, ethnicity, comorbidities), and health system factors (epidemiological surveillance systems, hospital resources, and access to health services). Factors related to the health system play a fundamental role in guaranteeing the opportunity and quality of care for patients with COVID-19 who require hospitalization(18). Social determinants of health have contributed to the hospital lethality rates from COVID-19 observed in different countries throughout the pandemic. Globally, the lethality observed by COVID-19 is less than 3%, although in countries like Mexico, it can reach values close to 10%(3). In this work, a lethality of 59.4% was observed in the period from March to September 2020, much higher than that reported globally for this type of population globally(19, 20), and than the COVID-19 lethality in Mexico in the same period (11%(21)). In Mexico, two studies carried out in tertiary hospitals in different geographical locations reported a lethality of 30.1%(4, 22).

The excess lethality observed in the present study may have several explanations, for example local factors in the health system. In this study, participants were enrolled in two public health institutions that serve different populations. The IMSS is a second-level federal public hospital that treated insured patients and had 180 beds available which reached a maximum occupancy of 130 beds and 75 mechanically ventilated patients. In contrast, the COVID-19 respiratory disease clinic is a public clinic-hospital managed by the Chiapas Ministry of Health, adapted to serve COVID-19 patients without any type of social security or healthcare coverage, had 20 enabled beds during the study period, and repeatedly reached maximum bed and intubation capacity. The saturation of both hospitals during the first outbreak in the state of Chiapas, as well as the capacity, infrastructure and medical personnel available to care for patients, could have directly influenced the observed lethality. In the univariate analysis, a statistically significant difference was found in the lethality between the two recruitment sites (IMSS 57% *vs* ISSTECH 79%; p = 0.028). However, in multivariate analyses this association was lost when the analysis was adjusted by individual conditions such as age, diabetes, and clinical variables. In Mexico, it has been estimated that lethality is higher (2 to 3 times) in public hospitals when compared to private hospitals(23). Likewise, delay in attention is thought to be a variable associated with COVID-19 lethality(24).

COVID-19 mortality risk has been consistently associated globally with certain biochemical markers, such as leukocyte and lymphocyte count, concentrations of proinflammatory interleukins and growth factors, levels of LDH, ferritin, and D-dimer(25, 26). Although in the univariate analysis several markers were consistent with associations previously reported in the literature (Table 2), when evaluated in the multivariate analysis, only age, diabetes, LDH and leukocyte count were associated with an increased chance of dying (Table 3). In general terms, our patients did not show a pattern of biochemical markers that explained the observed excess lethality.

Another contributing factor to the observed excess lethality was the infection severity at admission defined by the OSI-WHO, which assesses a patient’s need for supplemental oxygen, including mechanical ventilation and additional support. The need for the use of supplemental oxygen as a risk factor for death from COVID-19 has been discussed without reaching a consensus(27, 28). In our population, we analyzed the OSI-WHO scale(29) in the first 48 hours after hospital admission as a possible marker of mortality risk, and found a statistically significant association. The greater the need for oxygen, the greater the risk of dying from COVID-19 (Tables 1 and 3). This finding shows that patients who developed respiratory failure in the first 48 hours after admission had a higher risk of death, regardless of any other factor evaluated in these cohorts.

Unexpectedly, the male sex, which is one of the risk factors most commonly associated with mortality risk from COVID-19 globally(30, 31), was not a risk factor in our population (Table 1). We believe that the lack of differences in mortality by sex was due to the underlying deteriorated health of this population. For example, both men and women over 40 years of age (more than 94% of our cohort) had a higher prevalence of diabetes (W:37.2%, M:41.4%) than the population of the state of Chiapas (W:12.9%−18.2%, M:10.9%−13.3%), and than the Mexican population (W:14.3%−27.4%, M:11.9%−22.8%) of the same age groups (Supplementary table 2). Another example is renal insufficiency was higher in our study (W:7.0%, M:8.0%), than in Chiapas (W:1.4%−2.4%, M:0.7–1.3%) and Mexico (W:0.9%−2.4%, M:0.8%−2.1%) (Supplementary table 2)(32).

A limitation of this study is the lack of information on some variables such as oximetry, arterial gases, D-dimer, and weight/height to determine obesity, which have been found to be associated with death in other studies. Also, the small sample size limits the accuracy of association measure estimates and model performance.

## Conclusions

The factors that determine the COVID 19 mortality risk in a relatively healthy population are sex, age and comorbidities. However, as this study shows, when populations have underlying poor health, some of these factors lose their associations with risk, and others become more important. Further work is needed to determine the importance of this observation which if corroborated, may be a crucial element in public policy and epidemic outbreak management.

## Supplementary Material

Supplement 1

## Figures and Tables

**Table 1.** Baseline demographic characteristics of the COVID-19 patients, Tapachula, Mexico.

Characteristic	Overall n = 392	Alive n = 159	Dead n= 233	p-value

**Site of attention** n (%)				**0.028**
IMSS	323 (82.4)	139 (87.4)	184 (79)	
COVID Clinic ISSTECH	69 (17.6)	20 (12.6)	49 (21)	

**Sex** Male n (%)	263 (67.1)	107 (67.3)	156 (67)	0.943

**Age** years Mean (SD)	60 (13)	56 (13)	63 (12)	**<0.001**

**Days between symptom onset and admission** Median (IQR)	8 (5 – 11)	8 (6 – 11)	7 (5 – 11)	0.581

**Days of hospitalization**	9 (5 – 16)	8 (4 – 14)	10 (5 – 16)	0.916

**OSI-WHO Classification** n (%)				**<0.001**

Hospitalized, no oxygen therapy (4)	13 (3.3)	11 (6.9)	2 (0.9)	

Oxygen by mask or nasal prongs (5)	177 (45.2)	98 (61.6)	79 (33.9)	

Non-invasive ventilation or high-flow oxygen (6)	147 (37.5)	44 (27.7)	103 (44.2)	

Intubation and MV (7a)	28 (7.1)	3 (1.9)	25 (10.7)	

MV + organ support/ECMO (7b)	27 (6.9)	3 (1.9)	24 (10.3)	

**Fever** n (%)	339 (86.5)	144 (90.6)	195 (83.7)	**0.046**

**Cough**	312 (79.6)	121 (76.1)	191 (82)	0.159

**Dyspnea** ^a^	309 (79)	121 (76.1)	188 (81)	0.241

**Myalgia**	238 (60.7)	107 (67.3)	131 (56.2)	**0.027**

**Arthralgia**	228 (58.2)	101 (63.5)	127 (54.5)	0.075

**Headache**	206 (52.6)	91 (57.2)	115 (49.4)	0.125

**Deterioration of general condition**	167 (42.6)	68 (42.8)	99 (42.5)	0.956

**Chest pain**	115 (29.3)	48 (30.2)	67 (28.8)	0.760

**Diarrhea**	87 (22.2)	46 (28.9)	41 (17.6)	**0.008**

**Odynophagia**	87 (22.2)	42 (26.4)	45 (19.3)	0.098

**Irritability**	81 (20.7)	38 (23.9)	43 (18.5)	0.193

**Malaise**	83 (21.2)	43 (27)	40 (17.2)	**0.020**

**Rhinorrhea**	70 (17.9)	31 (19.5)	39 (16.7)	0.485

**Dysgeusia/Ageusia** ^b^	46 (11.8)	20 (12.7)	26 (11.2)	0.653

**Anosmia**	46 (11.7)	21 (13.2)	25 (10.7)	0.456

**Polypnea**	46 (11.7)	18 (11.3)	28 (12)	0.833

**Cyanosis**	18 (4.6)	3 (1.9)	15 (6.4)	**0.025**

**Hypertension**	161 (41.1)	60 (37.7)	101 (43.4)	0.267

**Diabetes**	157 (40.1)	54 (34)	103 (44.2)	0.041

**Chronical kidney disease**	30 (7.7)	9 (5.7)	21 (9)	0.212

a There was one missing value in death patients (n=232).

b There was one missing value in alive patients (n=158).

**Table 2.** Baseline blood test results and treatment of the COVID-19 patients, Tapachula, Mexico.

Characteristic	n	Overall n = 392	Alive n = 159	Dead n= 233	p-value
**Hemoglobin** GMT (LCL-UCL)	144/203	12.8 (12.6 – 13.1)	12.9 (12.5 – 13.4)	12.8 (12.5 – 13.1)	0.052
**Red cell distribution width**	140/198	14.3 (14.0 – 14.6)	13.9 (13.7 – 14.1)	14.6 (14.1 – 15.1)	**<0.001**
**Leukocyte count × 10^3^/cc**	144/203	11.9 (11.4 – 12.5)	10.6 (9.9 – 11.4)	13.0 (12.1 – 13.8)	**<0.001**
**Neutrophil count × 10^3^/cc**	144/202	10.0 (9.4 – 10.6)	8.6 (7.9 – 9.3)	11.1 (10.3 – 11.9)	**<0.001**
**Lymphocyte count × 10^3^/cc**	144/202	0.67 (0.61 – 0.73)	0.86 (0.77 – 0.96)	0.56 (0.50 – 0.63)	**<0.001**
**NT/LYN ratio**	144/201	15 (13 – 17)	10 (9 – 12)	20 (17 – 23)	**<0.001**
**Platelet count × 10^3^/cc**	144/203	271 (258 – 284)	283 (265 – 303)	262 (245 – 280)	0.13
**Prothrombin time** sec.	114/174	13.2 (13.0 – 13.4)	12.9 (12.6 – 13.1)	13.5 (13.2 – 13.8)	**<0.001**
**Activated partial thromboplastin time** sec.	112/170	34 (33 – 35)	33 (32 – 35)	34 (33 – 36)	0.4
**INR**	114/174	1.12 (1.11–1.14)	1.09 (1.06–1.11)	1.15 (1.13–1.18)	**<0.001**
**Glycemia** mg/dL	135/195	156 (146 – 167)	139 (126 – 153)	170 (155 – 185)	**<0.001**
**Urea** mg/dL	132/192	47 (43 – 52)	36 (32 – 42)	56 (50 – 63)	**<0.001**
**Creatinine** mg/ml	134/193	1.3 (1.1 – 1.4)	1.0 (0.9 – 1.2)	1.5 (1.3 – 1.7)	**<0.001**
**LDH** UI/L	128/176	515 (487 – 545)	432 (400 – 467)	585 (543 – 631)	**<0.001**
**Total bilirubin** mg/ml	134/186	0.64 (0.60 – 0.68)	0.64 (0.58 – 0.71)	0.64 (0.59 – 0.69)	>0.9
**ALT** UI/L	135/192	49 (45 – 53)	47 (42 – 52)	50 (45 – 56)	0.6
**AST** UI/L	135/191	42 (38 – 45)	45 (40 – 51)	39 (35 – 44)	**0.017**
**Albumin** g/dL	122/162	3.0 (2.9 – 3.1)	3.2 (3.0 – 3.3)	2.9 (2.8 – 3.0)	**<0.001**
**Sodium** mEq/L	127/180	134 (133 – 135)	135 (134 – 136)	134 (133 – 135)	>0.9
**Potassium** mmol/L	132/196	4.3 (4.2 – 4.4)	4.3 (4.1 – 4.4)	4.4 (4.3 – 4.5)	0.11
**Chlorine** mEq/L	132/196	98 (96 – 100)	100 (99 – 101)	96 (93 – 100)	0.14
**Any antibiotic** n%	155/226	335 (87.9)	135 (87.1)	200 (88.5)	0.681
**Ceftriaxone (CFT)**	155/226	280 (73.5)	117 (75.5)	163 (72.1)	0.464
**Azithromycin (AZT)**	155/226	264 (69.3)	108 (69.7)	156 (69)	0.892
**AZT and CFT**	155/226	221 (58)	92 (59.4)	129 (57.1)	0.658
**Steroid**	155/226	77 (20.2)	29 (18.7)	48 (21.2)	0.545

**Table 3.** Predictive models for COVID-19 death using logistic regression (training set: 70% of patients).

Characteristic	OR (95%CI)	M1 ORa (95%CI)	M1a ORa (95%CI)	M2 ORa (95%CI)

**Age**	1.04 (1.02–1.06)	**1.04 (1.02 – 1.07)**	**1.04 (1.02 – 1.07)**	**1.05 (1.02 – 1.07)**

**OSI-WHO Classification**				

Hospitalized, no oxygen therapy (4)	Ref.	Ref.	Ref.	

Oxygen by mask or nasal prongs (5)	5.97 (1.02 – 113)	4.67 (0.92 – 36.24)	4.14 (0.83–32.60)	

Non-invasive ventilation or high-flow oxygen (6)	16.2 (2.73 – 309)	**8.8 (1.66 – 69.87)**	**8.53 (1.65–68.70)**	

Intubation and MV (7a)	112 (9.48 – 4,403)	**26.92 (3.29–324.38)**	**32.15 (3.93–398.0)**	

MV + organ support/ECMO (7b)	59.5 (6.50 – 1,511)	**22.41 (2.63–276.85)**	**20.08 (2.46–242.46)**	

**Diarrhea**	0.47 (0.26 – 0.85)	0.496 (0.24 – 1.01)	0.55 (0.27 – 1.10)	**0.44 (0.22 – 0.85)**

**Diabetes**	2.05 (1.23 – 3.46)	**1.97 (1.05 – 3.78)**		

**Log 2 LDH** UI/L	2.33 (1.52 – 3.74)	**2.95 (1.66 – 5.49)**	**3.05 (1.78 – 5.51)**	**4.35 (2.63 – 7.60)**

**Log 2 AST** UI/L	0.77 (0.60 – 0.98)	**0.58 (0.42 – 0.81)**	**0.54 (0.40 – 0.74)**	**0.58 (0.43 – 0.77)**

**Log2 Lymphocyte**	0.62 (0.47 – 0.81)	**0.495 (0.31 – 0.82)**	**0.50 (0.32 – 0.79)**	**0.55 (0.41 – 0.72)**

**Log2 Leukocyte count**	1.69 (1.14 – 2.55)	**1.94 (1.10 – 3.44)**	**1.77 (1.03 – 3.01)**	

**NT/LYN ratio**	1.02 (1.01 –11.04)	0.99 (0.98 – 1.01)	0.99 (0.98 – 1.01)	

**Log2 Platelet count**	0.62 (0.40 – 0.92)	0.70 (0.42 – 1.17)		

**Log 2 Red cell distribution width**	7.11 (1.36 – 55.2)	7.66 (1.13 – 87.66)		

**Site of attention**				
IMSS	Ref.	Ref.	Ref.	Ref.
COVID Clinic ISSTECH	1.46 (0.77 – 2.86)	0.83 (0.26 – 2.67)	0.68 (0.23 – 2.0)	0.78 (0.29 – 2.17)

**Table 4.** Model performance in treating set (Tr, n=276) and test set (T, n=116).

Model	Accuracy	Sensitivity	Specificity	PPV	NPV	AUC (95%CI)
Model M1 (Tr)	0.756	0.792	0.706	0.792	0.706	0.807 (0.745 – 0.869)
Model M1a (Tr)	0.761	0.825	0.671	0.780	0.731	0.806 (0.744 – 0.868)
Model M2 (Tr)	0.751	0.783	0.706	0.790	0.698	0.791 (0.726 – 0.856)
Model M1 (T)	0.762	0.818	0.700	0.750	0.778	0.764 (0.671 – 0.857)
Model M1a (T)	0.744	0.737	0.759	0.857	0.595	0.726 (0.632 – 0.820)
Model M2 (T)	0.767	0.774	0.758	0.837	0.676	0.756 (0.664 – 0.849)

## Abbreviations

AIC: Akaike information criterion
AST: Aspartate aminotransferase
AUC: Area under the ROC Curve
BIC: Bayesian information criterion
CBC: Complete blood count
CDC: Centers for Disease Control and Prevention
COVID-19: Coronavirus disease 2019
CRF: Case report form
ECMO: Extracorporeal membrane oxygenation
IMSS: Instituto Mexicano del Seguro Social
INR: International normalized ratio
ISSTECH: Instituto de Seguridad Social de los Trabajadores del Estado de Chiapas
LDH: Lactate dehydrogenase
NT/LY ratio: Neutrophil/Lymphocyte ratio
OSI-WHO Classification: Ordinal scale of clinical improvement of the World Health Organization
PPV: Positive predictive value
NPV: Negative predictive value
ROC: Receiver operating characteristic curve
RT-qPCR: Quantitative reverse transcription polymerase chain reaction
WHO/PAHO: World Health Organization/Pan American Health Organization
