# Supplementary material for: Risk Prediction of Death in Inpatient Adults With COVID-19 from Mexico
Source: Res Sq. 2021 Nov 2:rs.3.rs-996535. Preprint. [Version 1] doi: 10.21203/rs.3.rs-996535/v1 (PMC8575141; doi:10.21203/rs.3.rs-996535/v1)
Supplement: Supplement 1 [file 023bead55acde6278d6922e0.docx]

Supplementary Table 1. Baseline characteristics and blood test results by sex

| **Characteristic** | **Overall**  **n = 392** | **Female**  **n = 129** | **Male**  **n= 263** | **p-value^a^** |  |
| --- | --- | --- | --- | --- | --- |
| **Dead** |  |  |  | >0.9 |  |
| Alive | 159 (40.6) | 52 (40.3) | 107 (40.7) |  |  |
| Dead | 233 (59.4) | 77 (59.7) | 156 (59.3) |  |  |
| **Site of attention** |  |  |  | >0.9 |  |
| IMSS | 323 (82.4) | 107 (82.9) | 216 (82.1) |  |  |
| COVID Clinic ISSTECH | 69 (17.6) | 22 (17.1) | 47 (17.9) |  |  |
| **Days between symptom onset and admission** Median (IQR) | 8 (5- 11) | 7 (4 - 10) | 8 (5 - 11) | 0.14 |  |
| **Age** Median (IQR) | 61 (51 - 70) | 62 (52 - 71) | 61 (51 - 69) | 0.4 |  |
| **Days of hospitalization** Median (IQR) | 9 (5 - 16) | 9 (5 - 17) | 9 (4 - 16) | 0.5 |  |
| **OSI-WHO Classification** n% |  |  |  | 0.5 |  |
| Hospitalized- no oxygen therapy (4) | 13 (3.3) | 4 (3.1) | 9 (3.4) |  |  |
| Oxygen by mask or nasal prongs (5) | 177 (45.2) | 66 (51.2) | 111 (42.2) |  |  |
| Non-invasive ventilation or high-flow oxygen (6) | 147 (37.5) | 41 (31.8) | 106 (40.3) |  |  |
| Intubation and MV (7a) | 28 (7.1) | 10 (7.8) | 18 (6.8) |  |  |
| MV + organ support/ECMO (7b) | 27 (6.9) | 8 (6.2) | 19 (7.2) |  |  |
| **Fever** n% | 339 (86.5) | 112 (86.8) | 227 (86.3) | >0.9 |  |
| **Cough** | 312 (79.6) | 102 (79.1) | 210 (79.8) | >0.9 |  |
| **Dyspnea** | 309/391 (79.0) | 99 (76.7) | 210/262 (80.2) | 0.5 |  |
| **Myalgia** | 238 (60.7) | 82 (63.6) | 156 (59.3) | 0.5 |  |
| **Arthralgia** | 228 (58.2) | 76 (58.9) | 152 (57.8) | >0.9 |  |
| **Headache** | 206 (52.6) | 75 (58.1) | 131 (49.8) | 0.15 |  |
| **Deterioration of general condition** | 167 (42.6) | 57 (44.2) | 110 (41.8) | 0.7 |  |
| **Chest pain** | 115 (29.3) | 36 (27.9) | 79 (30.0) | 0.8 |  |
| **Diarrhea** | 87 (22.2) | 25 (19.4) | 62 (23.6) | 0.4 |  |
| **Odynophagia** | 87 (22.2) | 27 (20.9) | 60 (22.8) | 0.8 |  |
| **Irritability** | 81 (20.7) | 23 (17.8) | 58 (22.1) | 0.4 |  |
| **Malaise** | 83 (21.2) | 19 (14.7) | 64 (24.3) | **0.040** |  |
| **Rhinorrhea** | 70 (17.9) | 20 (15.5) | 50 (19.0) | 0.5 |  |
| **Dysgeusia/Ageusia** | 46/391 (11.8) | 15/128 (11.7) | 31 (11.8) | >0.9 |  |
| **Anosmia** | 46 (11.7) | 14 (10.9) | 32 (12.2) | 0.8 |  |
| **Polypnea** | 46 (11.7) | 22 (17.1) | 24 (9.1) | **0.034** |  |
| **Abdominal pain** | 35 (8.9) | 11 (8.5) | 24 (9.1) | >0.9 |  |
| **Cyanosis** | 18 (4.6) | 11 (8.5) | 7 (2.7) | **0.019** |  |
| **Hiporexia** | 14 (3.6) | 6 (4.7) | 8 (3.0) | 0.4 |  |
| **Vomit** | 12 (3.1) | 5 (3.9) | 7 (2.7) | 0.5 |  |
| **Nausea** | 10 (2.6) | 4 (3.1) | 6 (2.3) | 0.7 |  |
| **Dizziness** | 9 (2.3) | 4 (3.1) | 5 (1.9) | 0.5 |  |
| **Precordial pain** | 6 (1.5) | 4 (3.1) | 2 (0.8) | 0.094 |  |
| **Diabetes** | 157 (40.1) | 48 (37.2) | 109 (41.4) | 0.5 |  |
| **Hypertension** | 161 (41.1) | 52 (40.3) | 109 (41.4) | >0.9 |  |
| **Chronical kidney disease** | 30 (7.7) | 9 (7.0) | 21 (8.0) | 0.9 |  |
| **Cardiovascular** | 14 (3.6) | 3 (2.3) | 11 (4.2) | 0.6 |  |
| **Asthma/COPD** | 14 (3.6) | 6 (4.7) | 8 (3.0) | 0.4 |  |
| **Neurological diseases** | 11 (2.8) | 2 (1.6) | 9 (3.4) | 0.4 |  |
| **Cancer** | 8 (2.0) | 4 (3.1) | 4 (1.5) | 0.4 |  |
| **Other Chronic Condition** | 29 (7.4) | 9 (7.0) | 20 (7.6) | >0.9 |  |
| **Ceftriaxone (CFT)** | 280/381 (73.5) | 98/125 (78.4) | 182/256 (71.1) | 0.2 |  |
| **Azithromycin (AZT)** | 264/381 (69.3) | 89/125 (71.2) | 175/256 (68.4) | 0.7 |  |
| **AZT and CFT** | 221/381 (58.0) | 78/125 (62.4) | 143/256 (55.9) | 0.3 |  |
| **Steroid** | 77/381 (20.2) | 20/125 (16.0) | 57/256 (22.3) | 0.2 |  |
| **Hemoglobin g/dL** | 347 | 116 | 231 |  |  |
| GMT  (LCL - UCL) | 12.8  (12.6 - 13.1) | 12.1  (11.7 - 12.5) | 13.2  (12.9 - 13.6) | **<0.001** |  |
| **Red cell distribution width** | 338 | 113 | 225 |  |  |
| GMT  (LCL - UCL) | 14.3  (14.0 - 14.6) | 14.1  (13.9 - 14.3) | 14.4  (13.9 - 14.8) | 0.2 |  |
| **Leukocyte count** **x 10^3^**/cc | 347 | 116 | 231 |  |  |
| GMT  (LCL - UCL) | 11.9  (11.4 - 12.5) | 11.1  (10.2 - 12.2) | 12.3  (11.6 - 13.1) | 0.069 |  |
| **Neutrophil count x 10^3^/cc** | 346 | 116 | 230 |  |  |
| GMT  (LCL - UCL) | 10.0  (9.4 - 10.6) | 9.1  (8.3 - 10.1) | 10.  (9.7 - 11.2) | **0.035** |  |
| **Lymphocyte count x 10^3^/cc** | 346 | 115 | 231 |  |  |
| GMT  (LCL - UCL) | 0.67  (0.61 - 0.73) | 0.81  (0.71 - 0.93) | 0.61  (0.54 - 0.68) | **<0.001** |  |
| **NT/LYN ratio** | 345 | 115 | 230 |  |  |
| GMT (LCL - UCL) | 15 (13 - 17) | 11 (10 - 14) | 17 (15 - 20) | **<0.001** |  |
| **Platelet count x 10^3^ /cc** | 347 | 116 | 231 |  |  |
| GMT (LCL - UCL) | 271 (258 - 284) | 284 (262 - 307) | 264 (249 - 281) | 0.3 |  |
| **Prothrombin time** sec. | 288 | 93 | 195 |  |  |
| GMT  (LCL - UCL) | 13.2  (13.0 - 13.4) | 13.1  (12.8 - 13.5) | 13.3  (13.1 - 13.5) | 0.2 |  |
| **Activated partial thromboplastin time** sec. | 282 | 91 | 191 |  |  |
| GMT (LCL - UCL) | 34 (33 - 35) | 35 (33 - 37) | 33 (32 - 34) | 0.10 |  |
| **INR** | 288 | 93 | 195 |  |  |
| GMT  (LCL - UCL) | 1.12  (1.11 - 1.14) | 1.12  (1.08 - 1.15) | 1.13  (1.11 - 1.15) | 0.3 |  |
| **Glycemia** mg/dL | 330 | 107 | 223 |  |  |
| GMT (LCL - UCL) | 156 (146 - 167) | 156 (138 - 177) | 156 (144 - 169) | 0.4 |  |
| **Urea** mg/dL | 324 | 104 | 220 |  |  |
| GMT (LCL - UCL) | 47 (43 - 52) | 40 (33 - 47) | 51 (46 - 57) | **0.010** |  |
| **Creatinine** mg/ml | 327 | 107 | 220 |  |  |
| GMT (LCL - UCL) | 1.3 (1.1 - 1.4) | 1.1 (0.9 - 1.3) | 1.4 (1.2 - 1.6) | **0.003** |  |
| **LDH** UI/L | 304 | 101 | 203 |  |  |
| GMT (LCL - UCL) | 515 (487 - 545) | 492 (445 - 544) | 527 (493 - 564) | 0.2 |  |
| **Total bilirubin** mg/ml | 320 | 104 | 216 |  |  |
| GMT  (LCL - UCL) | 0.64  (0.60 - 0.68) | 0.50  (0.45 - 0.55) | 0.72  (0.67 - 0.77) | **<0.001** |  |
| **ALT** UI/L | 327 | 107 | 220 |  |  |
| GMT (LCL - UCL) | 49 (45 - 53) | 42 (37 - 48) | 52 (48 - 58) | **<0.001** |  |
| **AST** UI/L | 326 | 107 | 219 |  |  |
| GMT (LCL - UCL) | 42 (38 - 45) | 34 (29 - 39) | 46 (42 - 51) | **<0.001** |  |
| **Albumin** g/dL | 284 | 93 | 191 |  |  |
| GMT (LCL - UCL) | 3.0 (2.9 - 3.1) | 3.1 (3.0 - 3.2) | 3.0 (2.9 - 3.1) | 0.8 |  |
| **Sodium** mEq/L | 307 | 102 | 205 |  |  |
| GMT (LCL - UCL) | 134 (133 - 135) | 135 (133 - 136) | 134 (133 - 135) | 0.7 |  |
| **Potassium** mmol/L | 328 | 109 | 219 |  |  |
| GMT (LCL - UCL) | 4.3 (4.2 - 4.4) | 4.2 (4.1 - 4.4) | 4.4 (4.3 - 4.5) | **0.020** |  |
| **Chlorine** mEq/L | 328 | 109 | 219 |  |  |
| GMT (LCL - UCL) | 98 (96 - 100) | 96 (90 - 103) | 99 (98 - 100) | 0.5 |  |
| ^a^Statistical tests performed: chi-square test of independence; Wilcoxon rank-sum test; Fisher's exact test | | | | | |

Supplementary Table 2. Diabetes, hypertension and renal insufficiency prevalence in Mexico and Chiapas, ENSANUT 2018.

| **Location** | **Sex** | **Age** | **n** | **Diabetes** | **IC95%** | **Hypertension** | **IC95%** | **Renal insufficiency** | **IC95%** |
| --- | --- | --- | --- | --- | --- | --- | --- | --- | --- |
| Mexico | Men | <40 | 8439 | 1.56 | 1.24 - 1.88 | 5.91 | 5.10 - 6.72 | 0.34 | 0.19 - 0.50 |
| Mexico | Men | 40-60 | 7332 | 11.85 | 10.72 - 12.99 | 17.24 | 15.92 - 18.56 | 0.8 | 0.47 - 1.14 |
| Mexico | Men | >60 | 3725 | 22.79 | 20.81 - 24.76 | 35.92 | 33.48 - 38.36 | 2.11 | 1.40 - 2.82 |
| Mexico | Women | <40 | 10,135 | 2.55 | 2.12 - 2.97 | 6.76 | 6.02 - 7.49 | 0.63 | 0.35 - 0.91 |
| Mexico | Women | 40-60 | 8,727 | 14.28 | 13.21 - 15.35 | 23.26 | 21.86 - 24.66 | 0.86 | 0.60 - 1.11 |
| Mexico | Women | >60 | 4,712 | 27.39 | 25.46 - 29.32 | 48.9 | 46.57 - 51.23 | 2.42 | 1.63 - 3.22 |
| Chiapas | Men | <40 | 255 | 1.67 | 0 - 3.52 | 5.15 | 1.92 - 8.39 | 0 |  |
| Chiapas | Men | 40-60 | 229 | 10.89 | 6.13 - 15.65 | 14.08 | 9.45 - 18.71 | 0.71 | 0 - 0.18 |
| Chiapas | Men | >60 | 102 | 13.34 | 6.26 - 20.41 | 27.98 | 18.26 - 37.70 | 1.3 | 0 - 3.11 |
| Chiapas | Women | <40 | 398 | 2.24 | 0.58 - 3.91 | 5.94 | 3.85 - 8.04 | 0.75 | 0 - 0.16 |
| Chiapas | Women | 40-60 | 289 | 12.88 | 8.02 - 17.75 | 29.19 | 22.94 - 35.44 | 2.36 | 0.40 - 4.33 |
| Chiapas | Women | >60 | 142 | 18.24 | 10.67 - 25.81 | 40.51 | 30.35 - 50.67 | 1.36 | 0 - 3.26 |

Supplementary Table 3. Baseline characteristics and blood test results by site

| **Characteristic** | **Overall**  **N = 392** | **IMSS**  **n = 323** | **COVID Clinic ISSTECH**  **n = 69** | ***p*-value^a^** |
| --- | --- | --- | --- | --- |
| **Dead** n (%) |  |  |  |  |
| Alive | 159 (40.6) | 139 (43.0) | 20 (29.0) | **0.032** |
| Dead | 233 (59.4) | 184 (57.0) | 49 (71.0) |  |
| **Days between symptom onset and admission** Median (IQR) | 5.0 (3.0 - 9.0) | 5.0 (3.0 - 8.0) | 8.0 (5.0 - 10.0) | 0.4378 |
| **Sex** n (%) |  |  |  | 0.889 |
| Female | 129 (32.9) | 107 (33.1) | 22 (31.9) |  |
| Male | 263 (67.1) | 216 (66.9) | 47 (68.1) |  |
| **Age** Median (IQR) | 61 (51 - 70) | 62 (51 - 70) | 60 (48 - 65) | 0.1054 |
| **Days of hospitalization** Median (IQR) | 9 (5, 17) | 10 (5 - 17) | 7 (4 - 13) | 0.0655 |
| **OSI-WHO Classification** n% | 27 (6.) | 18 (5.6) | 9 (13.0) | **<0.001** |
| Hospitalized- no oxygen therapy (4) | 13 (3.3) | 13 (4.0) | 0 (0.0) |  |
| Oxygen by mask or nasal prongs (5) | 177 (45.2) | 163 (50.5) | 14 (20.3) |  |
| Non-invasive ventilation or high-flow oxygen (6) | 147 (37.5) | 113 (35.0) | 34 (49.3) |  |
| Intubation and MV (7a) | 28 (7.1) | 16 (5.0) | 12 (17.4) |  |
| MV + organ support/ECMO (7b) | 27 (6.9) | 18 (5.6) | 9 (13.0) |  |
| **Fever** n (%) | 339 (86.5) | 288 (89.2) | 51 (73.9) | **0.002** |
| **Cough** | 312 (79.6) | 270 (83.6) | 42 (60.9) | **<0.001** |
| **Dyspnea** | 309/391 (79.0) | 255/322 (79.2) | 54 (78.3) | 0.871 |
| **Myalgia** | 238 (60.7) | 206 (63.8) | 32 (46.4) | **0.010** |
| **Arthralgia** | 228 (58.2) | 199 (61.6) | 29 (42.0) | **0.003** |
| **Headache** | 206 (52.6) | 183 (56.7) | 23 (33.3) | **0.001** |
| **Deterioration of general condition** | 167 (42.6) | 157 (48.6) | 10 (14.5) | **<0.001** |
| **Chest pain** | 115 (29.3) | 112 (34.7) | 3 (4.3) | **<0.001** |
| **Diarrhea** | 87 (22.2) | 79 (24.5) | 8 (11.6) | **0.024** |
| **Odynophagia** | 87 (22.2) | 83 (25.7) | 4 (5.8) | **<0.001** |
| **Irritability** | 81 (20.7) | 81 (25.1) | 0 (0.0) | **<0.001** |
| **Malaise** | 83 (21.2) | 64 (19.8) | 19 (27.5) | 0.193 |
| **Rhinorrhea** | 70 (17.9) | 65 (20.1) | 5 (7.2) | **0.009** |
| **Dysgeusia/Ageusia** | 46/391 (11.8) | 44/322 (13.7) | 2 (2.9) | **0.012** |
| **Anosmia** | 46 (11.7) | 43 (13.3) | 3 (4.3) | **0.038** |
| **Polypnea** | 46 (11.7) | 43 (13.3) | 3 (4.3) | **0.038** |
| **Abdominal pain** | 35 (8.9) | 31 (9.6) | 4 (5.8) | 0.484 |
| **Cyanosis** | 18 (4.6) | 18 (5.6) | 0 (0.0) | 0.052 |
| **Hiporexia** | 14 (3.6) | 13 (4.0) | 1 (1.4) | 0.480 |
| **Vomit** | 12 (3.1) | 8 (2.5) | 4 (5.8) | 0.237 |
| **Nausea** | 10 (2.6) | 6 (1.9) | 4 (5.8) | 0.080 |
| **Dizziness** | 9 (2.3) | 9 (2.8) | 0 (0.0) | 0.370 |
| **Precordial pain** | 6 (1.5) | 6 (1.9) | 0 (0.0) | 0.596 |
| **Diabetes** | 157 (40.1) | 128 (39.6) | 29 (42.0) | 0.787 |
| **Hypertension** | 161 (41.1) | 134 (41.5) | 27 (39.1) | 0.788 |
| **Chronical kidney disease** | 30 (7.7) | 24 (7.4) | 6 (8.7) | 0.802 |
| **Cardiovascular** | 14 (3.6) | 13 (4.0) | 1 (1.4) | 0.480 |
| **Asthma/COPD** | 14 (3.6) | 10 (3.1) | 4 (5.8) | 0.283 |
| **Neurological diseases** | 11 (2.8) | 9 (2.8) | 2 (2.9) | 1 |
| **Cancer** | 8 (2.0) | 7 (2.2) | 1 (1.4) | 1 |
| **Ceftriaxone (CFT)** | 280/381 (73.5) | 231/312 (74.0) | 49/69 (71.0) | 0.652 |
| **Azithromycin (AZT)** | 264/381 (69.3) | 222/312 (71.2) | 49/69 (60.9) | 0.112 |
| **AZT and CFT** | 221/381 (58.0) | 183/312 (58.7) | 38/69 (55.1) | 0.593 |
| **Steroid** | 77/381 (20.2) | 60/312 (19.2) | 17/69 (24.6) | 0.322 |
| **Other Chronic Condition** | 29 (7.4) | 21 (6.5) | 8 (11.6) | 0.201 |
| **Hemoglobin g/dL** | 347 | 294 | 53 |  |
| GMT (LCL - UCL) | 12.84 (12.58 - 13.11) | 12.95 (12.69 - 13.22) | 12.27 (11.43 - 13.17) | 0.4 |
| **Red cell distribution width** | 338 | 287 | 51 |  |
| GMT (LCL - UCL) | 14.28 (13.99 - 14.58) | 14.34 (14.01 - 14.68) | 13.96 (13.50 - 14.43) | 0.2 |
| **Leukocyte count** **x 10^3^**/cc | 347 | 294 | 53 |  |
| GMT (LCL - UCL) | 11.9 (11.4 - 12.5) | 11.8 (11.2 - 12.5) | 12.4 (10.9 - 14.1) | 0.6 |
| **Neutrophil count x 10^3^/cc** | 346 | 294 | 52 |  |
| GMT (LCL - UCL) | 10.0 (9.4 - 10.6) | 9.9 (9.2 - 10.5) | 10.6 (9.1 - 12.2) | 0.5 |
| **Lymphocyte count x 10^3^/cc** | 346 | 293 | 53 |  |
| GMT (LCL - UCL) | 0.67 (0.61 - 0.73) | 0.64 (0.59 - 0.70) | 0.83 (0.63 - 1.10) | **0.004** |
| **NT/LYN ratio** |  |  |  |  |
| GMT (LCL - UCL) | 15 (13 - 17) | 15 (14 - 17) | 13 (9 - 18) | 0.068 |
| **Platelet count x 10^3^ /cc** | 347 | 294 | 53 |  |
| GMT (LCL - UCL) | 271 (258 - 284) | 270 (256 - 284) | 275 (243 - 311) | >0.9 |
| **Prothrombin time** sec. | 288 | 246 | 42 |  |
| GMT (LCL - UCL) | 13.24 (13.04 - 13.43) | 13.38 (13.17 - 13.60) | 12.42 (12.02 - 12.84) | **<0.001** |
| **Activated partial thromboplastin time** sec. | 282 | 244 | 38 |  |
| GMT (LCL - UCL) | 34 (33 - 35) | 34 (33 - 35) | 34 (32 - 37) | 0.6 |
| **INR** | 288 | 246 | 42 |  |
| GMT (LCL - UCL) | 1.12 (1.11 - 1.14) | 1.12 (1.10 - 1.14) | 1.14 (1.08 - 1.21) | >0.9 |
| **Glycemia** mg/dL | 330 | 272 | 58 |  |
| GMT (LCL - UCL) | 156 (146 - 167) | 152 (141 - 164) | 175 (153 - 201) | 0.10 |
| **Urea** mg/dL | 324 | 268 | 56 |  |
| GMT (LCL - UCL) | 47 (43 - 52) | 46 (42 - 51) | 53 (44 - 65) | 0.4 |
| **Creatinine** mg/ml | 327 | 270 | 57 |  |
| GMT (LCL - UCL) | 1.3 (1.1 - 1.4) | 1.2 (1.1 - 1.4) | 1.4 (1.1 - 1.9) | 0.9 |
| **LDH** UI/L | 304 | 267 | 37 |  |
| GMT (LCL - UCL) | 515 (487 - 545) | 479 (454 - 505) | 870 (720 - 1-052) | **<0.001** |
| **Total bilirubin** mg/ml | 320 | 268 | 52 |  |
| GMT (LCL - UCL) | 0.64 (0.60 - 0.68) | 0.67 (0.63 - 0.71) | 0.50 (0.43 - 0.59) | **0.004** |
| **ALT** UI/L | 327 | 270 | 57 |  |
| GMT (LCL - UCL) | 49 (45 - 53) | 49 (45 - 53) | 49 (38 - 63) | 0.4 |
| **AST** UI/L | 326 | 269 | 57 |  |
| GMT (LCL - UCL) | 42 (38 - 45) | 39 (36 - 43) | 55 (44 - 70) | **0.019** |
| **Albumin** g/dL | 284 | 264 | 20 |  |
| GMT (LCL - UCL) | 3.03 (2.94 - 3.12) | 3.03 (2.95 - 3.11) | 3.04 (2.47 - 3.74) | 0.074 |
| **Sodium** mEq/L | 307 | 266 | 41 |  |
| GMT (LCL - UCL) | 134 (133 - 135) | 134 (133 - 135) | 136 (134 - 138) | 0.078 |
| **Potassium** mmol/L | 328 | 271 | 57 |  |
| GMT (LCL - UCL) | 4.33 (4.24 - 4.41) | 4.36 (4.27 - 4.44) | 4.19 (3.95 - 4.44) | **0.010** |
| **Chlorine** mEq/L | 328 | 271 | 57 |  |
| GMT (LCL - UCL) | 98 (96 - 100) | 97 (95 - 100) | 99 (97 - 102) | 0.14 |
| ^a^Statistical tests performed: Wilcoxon rank-sum test; Fisher's exact test | | | | |
